# Cystic Fibrosis Year in Review 2024

**DOI:** 10.1002/ppul.71222

**Published:** 2025-08-08

**Authors:** Adrienne P. Savant

**Affiliations:** ^1^ Department of Pediatrics Manning Family Children's New Orleans Louisiana USA; ^2^ Department of Pediatrics Tulane University New Orleans Louisiana USA

**Keywords:** CFTR modulators, cystic fibrosis, newborn screening, pulmonary exacerbations

## Abstract

In 2024, important advances for people with cystic fibrosis (CF) were published. Important guidelines for newborn screening and care of infants diagnosed with CF transmembrane conductance regulator (CFTR)‐Related Metabolic Syndrome/Cystic Fibrosis Screen Positive Inconclusive Diagnosis (CRMS/CFSPID) were published alongside related key lessons from individual programs. Work continues to improve growth and nutrition and treat pulmonary exacerbations. New position papers on care delivery and the care team in the post‐CFTR modulator era were developed next to continued information related to CFTR modulator use on treatment burden simplification and side effects, such as mental health and use during pregnancy. The aim of this review is to provide high‐level information that may lead to changes in clinical care.

## 1 Introduction

The cystic fibrosis (CF) community has published many studies across a wide range of topics in 2024. This review will not be exhaustive of all publications related to CF from the past year but will focus on clinical topics related to newborn screening (NBS), CF transmembrane conductance regulator (CFTR)‐Related Metabolic Syndrome/Cystic Fibrosis Screen Positive Inconclusive Diagnosis (CRMS/CFSPID), pulmonary exacerbations, use of acid suppression, CFTR modulators, and new/updated guidelines. The information provided is aimed to provide the pertinent details of the research, without excessive detail, to facilitate high‐level conclusions; however, if a more comprehensive understanding of the research is desired, further information can be found through evaluation of the full manuscripts.

## 2 Newborn Screening

CF NBS has been implemented in all states in the United States (US), most European countries, and many Latin American countries. In the US, guidelines for CF NBS were published, with highlights outlined in Table 1 [1]. Critical to the success of NBS programs is the iterative process of cyclic feedback from multiple stakeholders (NBS program and CF clinicians) partnered with outcome‐related data [2]. Although implementation and protocols vary across the globe, lessons can be gained from the various programs.

**Table 1:** 2025 consensus guideline cystic fibrosis newborn screening [1].

The Cystic Fibrosis Foundation recommends:
Use of a floating IRT cutoff over a fixed IRT cutoff. Use a very high IRT referral strategy when variant panel does not include all CF‐causing variants in CFTR2.org or does not have a variant panel that achieves at least 95% sensitivity in all ancestral groups within the state Not limit *CFTR* variant detection to only F508del variant or variants included in the ACMG‐23 panel. Screen for all CF‐causing *CFTR* variants as identified by CFTR2.org Conduct *CFTR* variant screening twice weekly or more frequently as resources allow. Include a *CFTR* sequencing tier following IRT and *CFTR* variant panel testing to improve the specificity and positive predictive value. Notify both the primary care provider and the CF specialist of abnormal results.

Abbreviations: ACGM, American College of Medical Genetics; CFTR, cystic fibrosis transmembrane regulatory conductance; IRT, immunoreactive trypsinogen.

The NBS program in the state of New Jersey (NJ) is an example of how communication partnered with data can lead to reduced disparities and improved outcomes. Five cases of delayed diagnosis, after false negative NBS, had significant clinical consequences such as respiratory failure requiring intubation and bronchiectasis at a young age [3]. Two of the infants were non‐White, a higher proportion than would be expected based on the racial and ethnic makeup of individuals with CF in the US. The cases were shared with the NBS program to advocate for changes in the protocol. Through this feedback from the CF Centers to the state lab, the NJ NBS program implemented changes in their protocol to improve equity, such that they lowered their immunoreactive trypsinogen (IRT) from ≥ 90 to ≥ 70 ng/mL and expanded variant panel from F508del only to 139 variants. This example also highlights one of the key findings from a qualitative study of key CF NBS informants, the importance of this communication between the state NBS lab and CF Centers regarding results of diagnostic testing and any delayed identification due to false negative results as part of a critical assessment of the CF NBS system [2].

One important area for NBS programs to review is related to racial and ethnic disparities. From 2010 to 2018, 21% of newly diagnosed patients in the US CF Foundation Patient Registry (CFFPR) were from minoritized racial and ethnic groups. Despite this, when 383 caregivers of children with CF (CwCF) were surveyed, 20% said their race, ethnicity, or family background was mentioned as making it unlikely their child would have CF (while 23% mentioned this in relation to likely to have CF) [4]. There continues to be a misconception of CF only affects white groups. Advocacy and education from CF Centers to the NBS lab to correct this erroneous belief are important in the steps toward equity. NBS labs should be aware that disparities exist in *CFTR* variant panels, such that some panels are not equitably finding one or more variants within the NBS programs specific population. As an example, the American College of Medical Genetics 23 and Illumina 39 only identify one variant in 73.4% and 83.4% of Black individuals compared to 95.3% and 96.7% in White individuals, respectively [2].

Despite the expanding number of variants on DNA panels for CF NBS, CwCF are still missed, thus, some NBS programs include sequencing. California has included CFTR sequencing since 2007 using a tiered approach [5]. IRT in the top 1.6% of values will have a *CFTR* variant panel run (varied over time from 28 to 75 variants). If only one variant is found, CFTR sequencing occurs, with only those tests with two variants found being called positive. A review of outcomes through 2021 showed 84 infants diagnosed with CF despite negative NBS (0.001% of total screened population and 9.2% of known CF population born in same time period). False negative NBS were disproportionately higher in nonwhite races and ethnicities. Of those missed infants, 45% (*n* = 38) were due to a low IRT, and 55% (*n* = 46) were due to only having 0 or 1 variant. Overall, although California was able to demonstrate an increased identification of two *CFTR* variants in all races and ethnicities, they noted that false‐negative screening was still more likely in racial and ethnic minorities.

As extended genetic sequencing is implemented an unintended consequence is the identification of more inconclusive results. In a survey of 108 health care professionals around the world, 80% felt it was more important to have a sensitive approach resulting in fewer CwCF being missed, resulting in more CRMS/CFSPID [6]. CRMS/CFSPID is defined as people with an abnormal CF NBS result and (1) a sweat chloride of < 30 mmol/L and 2 *CFTR* variants, at least one of which has unclear phenotypic consequences or (2) a sweat chloride of ≥ 30 to 59 mmol/L and one or no CF‐causing variants [7]. The authors note that this differs from the general public, which has been reported to favor a more specific approach (more CwCF being missed and less CRMS/CFSPID).

As CF NBS continues to be evaluated, strong communication between the care centers and NBS programs is critical to ensure that equitable, timely diagnoses are made in all populations. CF Centers and NBS programs should review critical data/cases on an ongoing basis. The NBS guidelines provide a creditable framework for improving CF NBS; however, continued work to refine ideal protocols that reduce disparities while optimizing clinical outcomesis still needed.

## 3 CRMS/CFSPID

A consequence of CF NBS is the diagnosis of CRMS/CFSPID. The natural history of these individuals is an area of great study as a balance between ensuring adequate clinical care and not over medicalizing these individuals is important. Over time, much evidence has been elucidated and in 2024 the US published evidence‐based management guidelines with the key recommendations briefly summarized in Table 2 [7].

**TABLE 2:** Cystic fibrosis foundation evidence‐based guideline for the management of CRMS/CFSPID [2].

The Cystic Fibrosis Foundation brief recommends for people with CRMS/CFSPID:	
Genetics	Consider sequencing and deletion/duplication analysis of coding and flanking regions and in select cases sequencing of intronic regions
	Consider genetic evaluation for parents when phasing the *CFTR* variants (i.e., in cis or trans)
	Offering CFTR genetic evaluation for siblings
	Provide genetic counseling by a clinician with training or clinical expertise in CF and genetics with a genetic counselor accessible for further support
Monitoring	At least annual follow‐up by a CF clinician and nurse
	Repeat sweat chloride test at 6 months of life and annually, at least until age 8 years
	Selectively offer respiratory cultures
	Measure fecal elastase at the initial assessment and when clinically indicated
	Do not obtain routine laboratory evaluations
	Do not obtain routine pulmonary function testing or chest radiographs
Treatment	Implement standard CF infection prevention and control guidelines
	Selectively offering antibiotics, such as inhaled antibiotics for *Pseudomonas aeruginosa* or oral antibiotics for unexplained prolonged cough (>2 weeks)
	Nutritional management by primary care providers if adequate growth; however, if a downward weight trajectory, should be screened and evaluated by an experienced dietician
	Do not provide salt supplementation or fat‐soluble vitamins
	Do not use routine airway clearance, and if new respiratory symptoms, use selectively
	Do not provide CFTR modulators
	Insufficient evidence for or against medications usually used to treat CF respiratory symptoms
Psychosocial and communication	Assess and consider social determinants of health that can influence the understanding and psychological impact of diagnosis and tailor communications appropriately
	Provide health care providers with accurate and up‐to‐date education about CRMS/CFSPID, its management, and the state's newborn screen program
	Depression and anxiety screening: offer at least one primary caregiver annually and individuals over age 12 years

Abbreviations: CFTR, cystic fibrosis transmembrane regulatory conductance; CRMS/CFSPID, Cystic Fibrosis Related Metabolic Syndrome/Cystic Fibrosis Screen Positive Inconclusive Diagnosis

Variability in the follow‐up for individuals with CRMS/CFSPID exists. In New York (NY) State, as the NBS process changed to include sequencing after an initial DNA panel, the CRMS‐CFSPID cases increased [8]. Retrospective evaluation of these cases revealed variability in repeat sweat testing (median 52% (range 22%–100%)), fecal elastase (median 46% (range 22%–93%)), meeting with genetic counselor (median 24% (range 0%–98%)), and oropharyngeal culture (median 62% (range 11%–87%)). A similar evaluation in Italy, using a questionnaire, examined management of CRMS/CFSPID in a real‐world setting also revealed significant variation in adherence to guidelines [9]. Similar to NY, 56.5% of centers monitored fecal elastase or conducted sweat tests every 12 months, and 74% monitored respiratory cultures; however, genetic counseling was offered more frequently at 83% of centers. To address this variability, NY developed a quality improvement initiative to ensure repeat sweat testing, specifically in lost to follow up infants around the time of the COVID‐19 pandemic [10]. Sweat tests were repeated in 17% of the infants, and consensus recommendations were developed within NY for ongoing management of CRMS/CFSPID infants.

For infants with CRMS/CFSPID, ongoing analysis of longer‐term outcomes is also pertinent clinically, as it can help to inform conversations with families. Several NBS programs shared data related to their programs. NY state found that 29/223 infants subsequently had a sweat chloride elevation of ≥ 5 mmol/L; however, none of the infants had sweat chloride ≥ 60 mmol/L within the first 3 years [11]. In California, between 2007 and 2019, 1951 positive NBS CRMS/CFSPID were diagnosed in 64% (*n* = 1257). Transition from CRMS/CFSPID to CF occurred in 5.3% (95% CI 4.2%–7.1%) (*n* = 66), with almost two‐thirds due to changes in sweat chloride or fecal elastase. Analysis of IRT found that those who converted had higher median IRT values (91.5 vs. 82 ng/mL). In comparison, five centers in Italy had 329 infants diagnosed with CRMS/CFSPID between 2011 and 2019 and followed 81% (*n* = 268) for a mean of 5 years [12]. During that time, 10.8% (*n* = 29) transitioned to a diagnosis of CF based on sweat chloride values ≥ 60 mmol/L or multiorgan involvement. A small sampling of children with CRMS/CFSPID showed no differences in lung function testing (percent predicted forced expiratory volume in 1 s (ppFEV1) and lung clearance index (LCI)). Furthermore, a subset of children followed after 6 years of age revealed only 1% (1/80) transitioned from CRMS/CFSPID to CF, while 10% (10/80) transitioned from CFTR‐related disorder (CFTR‐RD) to CF, leading the authors to conclude that transition to CF after age 6 to be unlikely in those with a diagnosis of CRMS/CFSPID; however, evolution for those with CFTR‐RD was not negligible. In the US, an analysis of the CFFPR from 2010 to 2020 found that only 3.5% (42/1174) of infants diagnosed with CRMS/CFSPID transitioned to a diagnosis of CF [13]. It is important to note that defining a conversion to CF from CRMS/CFSPID has not been well defined in the past; thus, a sweat chloride elevation to > 60 mmol/L provides some clarity; the decision to change the diagnosis to CF is based on the clinical opinion of the provider. A comparison between those diagnosed with CF to those with CRMS/CFSPID after 9–10 years of age revealed the CRMS/CFSPID children had normal nutritional parameters (height/weight for age) and lung function (ppFEV1). Although CF‐specific infections were found in those with CRMS/CFSPID, the rates were significantly lower than in CF. Thus, for those diagnosed with CRMS/CFSPID, the transition to CF was uncommon, but not negligible, while health outcomes were essentially normal over time for those who did not convert.

## 4 Pulmonary Exacerbations

Although the rate of pulmonary exacerbations requiring IV antibiotics has significantly decreased with the use of CFTR modulators, people with CF (PwCF) who are ineligible to take modulators continue to have infections that lead to pulmonary exacerbations. When a pulmonary exacerbation is treated with IV antibiotics in the hospital, a subset of PwCF will fail to recover their lung function, leading clinicians to work to identify which individuals may not recover and what treatments may facilitate recovery.

To optimize recovery, adequate antibiotic coverage to treat known pathogens is important. To provide optimal treatment, microbiologic processing of respiratory samples is important. Based on advances in technology, awareness of testing that does not add additional clinical benefit and other research, new guidelines were published this year, and major updates are outlined in Table 3 [14].

**TABLE 3:** Practical guidance for clinical microbiology laboratories: Updated guidance for processing respiratory tract samples from people with cystic fibrosis [3].

Major updates to recommendations
More samples will be respiratory swabs due to less ability to expectorate sputum due to CFTR modulators—processing is the same as with sputum. Gram stains are not recommended to be routinely performed as use of a gram stain to reject samples is not predictive of potential pathogens. As expanded types of organisms have been detected in respiratory samples, it is important to detect and identify these less common gram‐negative bacteria and fungi to continue to facilitate epidemiology and understand their potential clinical significance. MALDI‐TOF (matrix‐assisted laser desorption ionization‐time of flight) provides excellent genus‐level identification; however, databases may not be representative of CF pathogens, so DNA sequencing should be available. Antimicrobial sensitivity testing should be limited to specific clinical circumstances, those impacting infection prevention and control, and antibiotic stewardship. Checkerboard synergy and multi‐combination bactericidal antibiotic testing are no longer recommended for multi‐drug resistance *Pseudomonas aeruginosa*. ∘ Methods to share patient clinical status ∘ Developing agreement for antimicrobial susceptibility testing ∘ Discussion of MALDI‐TOF limitations ∘ Indications for DNA sequence analysis

Abbreviations: CFTR, cystic fibrosis transmembrane regulatory conductance; DNA, deoxyribonucleic acid.

Prediction of failure to return to baseline lung function was seen when the Chronic Respiratory Infection Symptom Score (CRISS) was obtained within the first 24 h of admission for pulmonary exacerbation [15]. The CRISS scale is an eight‐item symptom burden questionnaire assessing difficulty breathing, feeling feverish, having chills/sweats, increased cough, increased mucus production, fatigue, chest tightness, and wheezing, with higher scores demonstrating higher symptom burden. The CRISS was evaluated as a secondary analysis of a prior study of 56 PwCF over the years 2007–2009. The authors concluded that for every increase in CRISS point, recovery of lung function decreased by 0.2% predicted points, such that a CRISS greater than 48.3, had a 14% greater risk of not recovering lung function by the end of treatment.

Steroids may be prescribed by clinicians to improve the likelihood of recovery of lung function. Using data from the standardized treatment of pulmonary exacerbations study (STOP2), where systemic steroids were allowed to be clinically prescribed, a sub‐analysis was conducted to assess the effect on recovery of lung function [16]. Initiation of steroids occurred primarily at two time points before, or post randomization. Earlier steroids use was associated with age ≥ 30 years, female, baseline ppFEV1 ≤ 50, and prior ABPA, while post randomization was solely associated with non‐early robust ppFEV1 response. Regardless of the time of initiation, systemic steroids did not result in improved ppFEV1, CRISS, C‐reactive protein, or weight.

Further understanding of the role of steroids in pulmonary exacerbations was examined in a 5‐year study at 14 centers in Canada from 2017 to 2022 [17]. Those individuals who had not responded with ppFEV1 recovery by Day 7 of IV antibiotics were randomized to receive steroids or placebo. Of the 80 non‐responders who were randomized, no difference was seen in ppFEV1 recovery by Day 14, Day 30, quality of life, CRISS, sputum inflammatory markers, number of days IV antibiotics, or time to next exacerbation.

Thus, as we continue to have PwCF who have pulmonary exacerbation, the use of the CRISS may help to identify those who are less likely to fully recover to their baseline lung function, and we can limit steroids due to a lack of a significant response.

## 5 Acid Supression

Growth is an important component of CF care, especially in individuals who are pancreatic insufficient and require pancreatic enzyme replacement therapy (PERT). Often acid suppression is used to help optimize PERT therapy; however, in recent years, negative outcomes related to acid suppression have been emerging, such as increased pulmonary infections, fractures from decreased bone density, and anemia. To analyze this further, a single‐center pilot, randomized, placebo‐controlled, cross over study evaluated the effect of proton pump inhibitors on PERT effectiveness [18]. PwCF, over age 12, with pancreatic insufficiency had a malabsorption blood test (MBT) obtained after 14 days of proton pump inhibitor or placebo. The MBT consisted of fasting for 12 h followed by a specific meal with known amounts of fatty acids that can be measured in the blood. Of the 13 PwCF who had MBT test, 78% had a decline in fatty acid absorption while taking the PPI, leading the authors to conclude a decrease in duodenal fatty acid absorption. Additionally, no changes were seen regarding gastric or small bowel transit times or quality of life measures.

Infants with CF who were on acid blocking treatment (proton pump inhibitor or H2 blocker) during the first 3 years of life from the Feeding Infants Right. From the Start (FIRST) study demonstrated no difference in nutritional outcomes (age/sex specific weight, height, and body mass index Z‐scores) [19]. However, those persistently on acid blocking therapy had worse early onset lung disease scores (reflecting respiratory symptoms, pulmonary exacerbations, *Pseudomonas aeruginosa* (PA) infections, and hospitalizations), with the majority of this difference occurring between 1 and 3 years of age, despite equal PA infections. Infants with gastrostomy tube feeds and antibiotic treatments also had increased early‐onset lung disease scores. Analysis of the gut microbiome demonstrated a reduction in the normal age‐related increases in diversity in those on persistent acid blocker therapy, especially from 2 to 3 years of age. Overall, the use of acid suppression should likely be considered individually with each patient to limit the negative consequences.

## 6 Modulator Therapy

Care for PwCF has drastically changed due to the use of CFTR modulators. Much has been learned about the new era of CF care leading to new ways to provide care. CFTR modulator usage has led to changes in CF epidemiology in a positive manner; however, use is also associated with side effects. As usage of these medications continues, more information is coming forth. This section will discuss updates to CF care in the modulator era.

### 6.1 Non Elexacaftor/Tezacaftor/Ivacaftor CFTR Modulator Studies

As there are several CFTR modulators available clinically, with CwCF under age 2 not eligible for elexacaftor/tezacaftor/ivacaftor (ETI) at this time, it is important to examine literature related to non‐ETI CFTR modulators. A couple of notable studies related to ivacaftor were published. The safety and tolerability of ivacator in CwCF 1‐4 months of age was demonstrated in an open‐label study over 24 weeks [20]. Pharmacokinetics behaved predictability, thus dosing is based on weight and age. Fecal elastase, IRT, and calprotectin were shown to be improved in participants. Pulmonary outcomes after ~3–4 years of ivacaftor was examined via data from the CFFPR, based on age of initiation of ivacaftor [21]. Those CwCF who started ivacaftor at 6–10 years of age, had a mean difference in ppFEV1 at 11–15 years of +6.31 (95% CI 2.63–9.78) compared to those who started 11–15 years of age. Similarly, findings occurred in 11–15‐year‐olds compared to 16–20‐year‐old (+11.22 (95% CI 6.96–16.47) and 16–20‐year‐old compared to 21–25‐year‐old (+5.89 (95% CI 0.75–11.72). Pulmonary exacerbations were also noted to be lower at early age of initiation, with a 52% lower exacerbation rate in those who initiated ivacaftor ages 6–10 compared to 11–15‐year‐old. These studies help to understand that CFTR modulator is safe in younger ages and leads to improved outcomes compared to later age of initiation; however, as use in young ages is still relatively new, the effect on more long‐term outcomes such as CF‐related diabetes or CF hepatobiliary disease has yet to be seen.

### 6.2 Redefining Care

As care has changed in the post‐CFTR modulator era, leading to two publications from the US CF Foundation, provided perspectives on redefining the CF care model [22] and the CF care team [23]. A brief outline of the key areas of change for the CF care model is provided in Table 4, with the evidence supporting these changes outlined in the position paper. The care team is based on a core team consisting of PwCF and caregivers supported by programmatic components and linked to essential partners with trained and trusted referrals. The key changes include additions to the core team of a pharmacist, mental health coordinator, and genetic counselor. Programmatic components include NBS, research coordination, sweat testing, microbiology, pharmacy support, and registry coordinators. Specific details are outlined in both publications to provide supporting guidance. In this new era of CF care, these two publications will help care centers to provide high level, consistent, quality care and assist in obtaining adequate support and resources for this care.

**TABLE 4:** Cystic Fibrosis Foundation position paper: Redefining the CF care model [4].

Areas of change:
Visit frequency: for ≥6 years of age, every 4–6 months can be determined when stable health with consideration for telemedicine in some situations Immunizations: addition of recommendation for the COVID‐19 vaccine and RSV monoclonal antibody Pulmonary function tests: for ≥6 years of age at least twice a year (with every visit) Imaging CXR: No benefit to use for surveillance CT Chest: Strongly encourage consideration in infants/preschoolers or any age in place of CXR, repeat at clinician discretion Abdominal Ultrasound: ≥3 years of age, every 2 years until adolescence, and baseline in adults

Abbreviations: CT, computed tomography; CXR, chest radiograph.

### 6.3 Mental Health

Since ETI was approved in the US in 2019, there have been conflicting data and variable reports related to associated changes in mental health, which were not seen with prior CFTR modulators. The context of these reports includes the baseline knowledge of elevated rate of mental health concerns (anxiety and depression) in individuals with CF and the timing coinciding with the COVID‐19 pandemic, where increased mental health concerns were seen in both PwCF and the general population. The reported changes in mental health ranged from improvements in depression symptoms to worsening mood, sleep concerns such as insomnia, increases in suicidality, and neurocognitive changes such as difficulty with word finding, “brain fog,” and memory and attention concerns. Reports of increased need for psychiatric medications have been published, alongside reports that improvements in mental health outcomes were seen in some PwCF with discontinuation or dose adjustment of ETI. Very few of the reports included pre‐ and post‐ETI assessments. Several proposed mechanisms for this include that CFTR is in the brain and is expressed in areas important for the regulation of mood and cognition; however, the exact role is not fully elucidated. Based on the above concerns, the 2024 ECFS standards of care statement recommended screening for changes in mental health during the first 3 months of treatment [24]. Additional literature from 2024 helped to further characterize the role of ETI in mental health changes.

Ramsey et al. systematically evaluated data for PwCF over age 6 years from clinical trials, post‐marketing reports, interim reports from post‐marketing safety studies, and peer‐reviewed literature, specifically assessing depression, suicidal ideation, attempts, and completion [25]. From the clinical trials data and post‐marketing reports, no difference in those on ETI compared to placebo was seen. Post marketing safety studies compared the 5 years before ETI use compared to 2 years post ETI and found no change in depression prevalence per year. Although there were numerous case reports and cohort studies, without a control population or comparison time period, it is hard to identify a causal role of ETI. From this systematic review of available data and reports, the authors concluded that depression related events with ETI were consistent with the background rate of these events in the CF population.

Using the national adverse drug reaction reporting system in the United Kingdom, the most common reaction for all modulators (2012–2023) was related to psychiatric effects at 13% [26], consisting of anxiety, depression, mood disturbances, sleep disturbances, and one fatality. A statistically significant increase in adverse drug reactions with psychiatric description was noted post ETI at 14.2% compared to 2.8% pre ETI, leading the authors to conclude that the increase adverse drug reactions in the psychiatric category was associated with the introduction ETI. Using the Federal Drug Administration Adverse Event Reporting System (FAERS) in the United States, all adverse event reports for ETI in individuals over 2 years of age from 2019 to 2024 were analyzed [27]. Similar to the UK, psychiatric complaints were the most frequently reported (odds ratio of 2.54), with elevations in anxiety, depression, and insomnia. These studies analyzed all ages together, therefore preventing an understanding of age‐related differences. Some of the literature from 2024 may start to tease apart the effect of different ages.

In an Australian adolescent population (10–18 years), a prospective, single center study of 31 PwCF, depression (the patient health questionnaire 9 (PHQ‐9)), anxiety (generalized anxiety disorder 7 (GAD 7)), and sleep disturbances (pediatric daytime sleepiness scale (PDSS)) were evaluated pre and post ETI initiation [28]. As the study was confounded by the COVID‐19 pandemic, the authors chose key pandemic related timepoints (baseline, 5th lockdown, lifting of lockdown, return to school, and 6 months post school return). There was no statistically significant change for either depression or anxiety, or sleep scores similar to Ramsey et al.

Expanding the age to school age and adolescents (8–17 years), a separate, single center, prospective longitudinal study used the PHQ‐8 and GAD‐7 in addition to the patient‐reported outcomes measurement information system‐anxiety and depression scores (PROMIS‐A and D) for up to 18 months post ETI initiation [29]. The authors found a decrease in elevated scores for depression (13% to 3% in PHQ8 and 8.8% to 2.6% in PROMIS‐D) and anxiety (8.9% to 0% in GAD 7% and 12.3% to 0% in PROMIS‐A). The authors recognized limitations due to small numbers (*n* = 81), use of only post‐ETI data, and no baseline.

In a younger cohort, a single center, longitudinal, short‐term evaluation of 6–11‐year‐olds used the pediatric symptom checklist (PSC) and SDSC at both baseline and 1 month post‐ETI initiation in 75 CwCF [30]. No statistically significant difference in the median total scores was seen, and similar percentages went from abnormal to normal scores compared with normal scores becoming abnormal for both scales (12% vs. 8% for PSC and 8% vs. 5% for SDSC).

In France, the youngest cohort, CwCF between 2 and 5 years of age, had behavioral, and sleep surveys assessed [31]. After 1 month of ETI usage, 47% (93/197) reported a sudden change in behavior, with 15% of those individuals (14/93) being an amplification of a pre‐existing condition. This behavior was described as ADHD (attention deficit hyperactivity disorder), irritability, and mood disorders. For 62% (58/93), this was present from the 1st week of ETI usage and persisted at 3 months. Immediate recovery was noted in 4 out of the 93 (4%) after there was either discontinuation or dose reduction of the ETI. Interestingly, the authors were able to obtain ETI plasma concentrations in 48 at 197 individuals and found no significant difference between those with or without new onset or amplify changes in behavior. They were also unable to find any changes related to age weight or sweat chloride response.

The above studies were primarily related to anxiety, depression, or sleep; however, neurocognitive effects have also been reported. In conjunction with the GAD‐7, PHQ‐9, and assessment of psychological side effects, the symbol digit modality test, a neurocognitive screener that assesses working memory and processing speed, was evaluated in 93 (20 adolescent ages) PwCF, over 12 years of age, pre ETI and up to 6 months post ETI [32]. No changes in the PHQ‐9 were noted, while the GAD‐7 had higher baseline scores compared to post scores, and psychological side effects were increased or stable over time. The symbol digit modality test increased over time after initiation of ETI.

Although adverse event reporting showed that the most common classification of reports was psychiatric in nature, there was no significant change in depression, anxiety, sleep disorders, or preliminary analysis of neurocognitive testing from the reported literature in 2024. The one outlier in these publications is in the youngest age group, 2–5‐year‐olds, where close to 50% of CwCF saw behavior changes after initiation of ETI, raising the concern about the effect on a more undeveloped, developing brain.

### 6.4 Metabolic Effects

Historically, PwCF have been thought to have minimal to no cardiac risk and had lower levels of total cholesterol (TC), high‐density lipoprotein (HDL), and low‐density lipoproteins (LDL) compared to their age‐matched peers, while elevations in triglycerides (TG), a known acute phase reactant, were often seen. Multiple single‐center studies evaluated the effect of CFTR modulators on cholesterol and lipid values after the introduction of ETI [29, 33, 34, 35, 36, 37, 38, 39]. All were in adults, with only two involving adolescents [29, 33]. Total cholesterol elevation was found to be statistically significant in all the studies. LDL was elevated in all but one study [33] while HDL was only found to be elevated in half of the studies [29, 36, 37]. Triglycerides were examined in a subset of the studies [29, 35, 36, 37, 38] with only one showing a significant elevation [36]. The proposed mechanism leading to alteration in these metabolic markers is likely related to decreased inflammation and increased appetite/absorption. Overall, it does appear that there is an elevation in total cholesterol, primarily due to LDL elevations, with HDL and TG remaining stable. It is important to monitor overtime to evaluate cardiac risk so that interventions can be implemented when indicated.

### 6.5 Infections

The effect of CFTR modulators on infections has not been fully characterized; however, it does appear there are decreases in the abundance of various infections and increased overall diversity, but eradication is unclear. Several publications looked further at infections from various perspectives. A longitudinal case series of 11 CwCF with long term PA, defined as five or more positive cultures, found that those not on ETI (*n* = 8) had approximately 5.8 courses of IV antibiotics/per child that required hospitalization over 3 years, while those on ETI (*n* = 3) had none despite continued long term PA infection [40]. Thus, despite continued isolation of PA, in this small case series, those on ETI had less morbidity. Another single‐center study found those with chronic PA (> 50% of their last 6 sputum cultures over at least 12 months were positive for PA) had the same clone when followed for 21 months post initiation of ETI, revealing persistence for some PA infections [41]. As many individuals on modulators no longer expectorate sputum, one site examined infections from sinuses in 38 PwCF over 42 months [42]. As with prior studies, they found a decreased abundance; however, there continued to be a persistence of PA and *Staphylococcus sp*. became more prominent. Sinus sampling did not consistently predict infections present in the sputum. A decreased prevalence was seen in PA, Methicillin Resistant *Staphylococcus aureus*, Methicillin Sensitive *Staphylococcus aureus*, *Stenotrophomonas maltophilia*, *Achromobacter xylosoxidans*, non‐tuberculous mycobacteria in a single center evaluation of 198 adults with cultures from 3 years before and 1 year post ETI [43]. No change was seen in *Hemophilous influenza* or *Burkholderia cenocepacia* specific for PA; there was a decrease in prevalence from 65% to 51%, a decrease in bacterial density in sputum, and no new colonization.

The Prospective Study to Evaluate Biological and Clinical Effects of Significantly Corrected CFTR Function (The PROMISE Study) included a PROMISE‐micro study that evaluated sputum from 177 PwCF at 27 centers before and for 3.5 years after initiation of ETI [44]. The study demonstrated a decrease in the prevalence of infections (PA, *Staphylococcus*, SM) that did not appear to rebound over time. In those individuals who had persistent infections after ETI, there was a decrease in culture density. Interestingly, for those PwCF who had negative cultures (no positive culture for 2 years before starting ETI), new infections occurred (36.4% (8/22) *Staphylococcus*, 11.9% (8/67) PA, and 10.3% (12/116) SM), although they were transient and at a low density. The authors point out that the relationship between a lower density or transient infections and lung disease in individuals on ETI is not clear. Overall, recent publications have demonstrated that with the use of CFTR modulators, although some infections persist, there is less infectious burden through reduced density and prevalence and lower clinical burden; however, new infections occur, albeit transiently and with lower density.

### 6.6 Discontinuation of Therapies

As individuals on CFTR modulators improve clinically, many are asking what amount of chronic therapy is indicated? The SIMPLIFY clinical trial evaluated the effect of short‐term (6 weeks) discontinuation of either dornase alfa or hypertonic saline in PwCF on ETI in PwCF over 12 years of age. As this study was unique due to being a nontherapeutic, discontinuation study, the QUEST (Qualitative Understanding of Experiences in the SIMPLFY Trial) explored participant experiences [45]. PwCF experienced night and day changes in their health after ETI, with a decreased cognitive burden from living with CF and a shift to managing overall wellness and comorbidities, leading to raised expectations for their overall health and an alteration in their self‐identity. The strong research community in CF facilitated participation in the SIMPLIFY study due to a strong trust in research teams, a sense of altruism, consideration of personal benefit, low protocol burden, and overall familiarity with research.

Additional clinical outcomes from the SIMPLIFY study demonstrated that sequential discontinuation of both agents was non inferior to the continuation of both therapies when evaluated by ppFEV1p and LCI _2.5_ [46]. It is important to note that extrapolation to PwCF with advanced disease or younger CwCF is limited, as participants had an average ppFEV1 of 97% and were older than 12 years of age at study entry. The authors appropriately point out that both medications are still key for CF, transitioning to intermittent therapy as compared to maintenance medications. A subset of participants in the SIMPLIFY study also had evaluation of their mucociliary clearance, using the average rate of whole right lung clearance through 90 min (WLAve90) as the primary outcome [47]. There was no difference in mucociliary clearance between those that continued versus discontinued hypertonic saline. Those who discontinued dornase alfa, demonstrated a 41% relative increase in mucociliary clearance (6.1% absolute change) over baseline, therefore favoring discontinuation.

An analysis of the cost savings of SIMPLIFY found an annual cost savings to the US health care system of $1.21 billion for dornase alfa and a more modest level decrease from hypertonic saline [48]. Even more important was the impact on individual treatment burden for PwCF, such that post‐SIMPLIFY follow‐up surveys showed that those PwCF randomized to discontinue medication were 8.7 and 5.2 times more likely to remain off dornase alfa and hypertonic saline, respectively [49].

Two additional publications examined medication use changes. One single‐center study in the US examined medication possession ratios in over 200 adolescent and adults with CF specifically for dornase alfa, hypertonic saline, and PERT [50]. Another publication used the Danish CF Registry, examined over 200 adolescents and adults for various classes of medications, such as airway and gastrointestinal [51]. Both found a significant decline in airway medications, with the US also demonstrating no decline in ppFEV1 despite reduced usage. Neither report demonstrated a decline in gastrointestinal medications compared to pre ETI. Overall, clinicians may consider using shared decision making, in appropriate populations when considering discontinuation of dornase alfa and/or hypertonic saline to decrease medical burden while continuing to ensure optimal pulmonary health, such as consideration of intermittent usage.

### 6.7 Pregnancy and Infants

Published case reports and series have provided information on the use of CFTR modulators during pregnancy in several populations: first, women with CF who are pregnant with infants not affected by CF, second, women with CF who are pregnant with infants affected by CF, and third, women without CF pregnant with a fetus with CF who take CFTR modulators off label to provide potential benefit to the fetus.

In women with CF who are pregnant, data from 11 US adult centers between 2010 and 2021 compared pregnancies on highly effective modulator therapy (HEMT) (*n* = 77) compared to pregnancies not on HEMT (*n* = 193) [52]. Data was collected 12 months before and 12 months after pregnancy. The ppFEV1 declined by −2.50 in those not on HEMT compared to an increase of 2.42 in those on HEMT. Although no difference was observed in infant outcomes, all infants born to mothers with CF were more likely than the general public to have been born via cesarean sections (47% vs. 20%–30%), preterm (33% vs. 10%), have a lower birth rate (21% vs. 10%) and more stays in NICU (30% vs. 8%–12%).

In women with CF carrying infants also affected by CF, reports have also provided benefits. A case series in one woman on ETI, highlighted benefits to the outcomes for two infants with CF [53]. Both infants were born with normal fecal elastase levels; however, the mother was unable to breastfeed due to other medicine contraindications, and the fecal elastase levels decreased over the first few months. Of interest, her male infant was found to have a normal appearance for age of the vas deferens through at least 3 months of age (infant was 10 months of age at time of publication).

In women who are carriers for a *CFTR* variant, ETI has also been used off‐label for infants affected by CF with ultrasound evidence of potential intestinal blockage (echogenic bowel) [54]. In three cases, ETI was utilized with varied outcomes. The mother used ETI for 8 weeks, from 31 weeks gestation to birth, and infant had no meconium ileus. Mother two used ETI 8 weeks, from 30 weeks gestation through birth, and the infant had some meconium plugs at birth. The third case involved a mother who started ETI at 35 weeks gestation and continued for 3 weeks until birth, and the infant required an exploratory laparotomy. None of the infants had cataracts when evaluated in the neonatal period, yet one had some cholestasis felt to be due to total parenteral nutrition and CF, not ETI. Another case series evaluated two infants with ETI exposure during pregnancy and continued exposure via breast milk [55]. No evidence of cataracts was seen in either of the two infants; however, one infant had a very low elevation of AST that remained stable, and the other infant had elevated LFTs that normalized by 9 months of age.

Thus, overall, the use of ETI during pregnancy has improved overall lung function for pregnant mothers with CF, potentially allowed for the resolution of meconium ileus, and has demonstrated a low burden of side effects in exposed infants. As there is insufficient evidence related to pregnancy‐related toxicity of CFTR modulators and it is off‐label in mothers carrying infants with CF, these clinical cases provide interesting insights; however, the risk is still present and for some payors, may not be covered in various situations and can be a large cost burden that will reduce equity in availability. It is still important to individualize discussions related to ETI exposure in pregnancy and through breast milk, to weigh risk and benefits with the individual using a shared decision‐making process, since high‐level evidence is not available.

### 6.8 Disparities

PwCF of racial and ethnic minorities are less likely to be eligible for CFTR modulators, and if eligible, have delayed access to CFTR modulators [56]. In a review of the CFFPR, those eligible for ETI over age 12 years between 2019 and 2022, demonstrated significant delays in ETI access for those of Black race, Hispanic ethnicity, and non‐private insurance. Median days to ETI prescription was prolonged at 177 days (95% CI 140, 221) and 133 days (95% CI 126, 142) in Black and Hispanic PwCF, compared to 120 days (95% CI 119, 122) in non‐Hispanic White PwCF. A separate analysis of the CFFPR for PwCF over age 6 years from 2020 to 2022, showed a similar result with ETI initiation being significantly lower in all other races and ethnicities compared to Non‐Hispanic Whites [57]. The time to 50% of eligible PwCF with a prescription for ETI was 112 days for non‐Hispanic Whites, 133 days for Asians, 127 days for Hispanics, 135 days for Black, and 122 days for other races. This delay persisted even after adjusting for age, lung function, insurance, and access to care centers. Further work needs to be undertaken to understand the reason for these disparities so that the barriers can be overcome to provide equality.

## 7 Conclusion

It is an exciting time for CF care with a new era leading to hope and decreased medical burden for the majority of individuals with CF, as shared in the selected publications from 2024. Guidelines have been developed/updated for handling respiratory specimens, NBS, and outlining of the new care team and care model. Although CFTR modulators are changing the CF paradigm, some aspects of CF care persist, such as pulmonary exacerbations and NBS. Throughout all of this study, disparities persist and work to improve equity remains. This review focused on clinically relevant topics; however, much is underway to bring new treatment modalities to PwCF who are not eligible for CFTR modulators such that research is not complete until there is a cure found for all those with CF.

## Author Contributions

**Adrienne P Savant:** conceptualization, writing – original draft, writing – review and editing.

## Conflicts of Interest

The author declares no conflicts of interest.

## Data Availability

Data sharing is not applicable to this article as no datasets were generated or analyzed during the current study.
